# O6-methylguanine-DNA methyltransferase (MGMT) Promoter methylation is a rare event in soft tissue sarcoma

**DOI:** 10.1186/1748-717X-7-180

**Published:** 2012-10-30

**Authors:** Jens Jakob, Maren Hille, Christian Sauer, Philipp Ströbel, Frederik Wenz, Peter Hohenberger

**Affiliations:** 1 Division of Surgical Oncology & Thoracic Surgery, Department of Surgery, University Medical Center Mannheim, University of Heidelberg, Th.-Kutzer-Ufer 1-3, Mannheim, 68137, Germany; 2 Department of Pathology, University Medical Center Mannheim, University of Heidelberg, Th.-Kutzer-Ufer 1-3, Mannheim, 68137, Germany; 3 Department of Radiation Oncology, University Medical Center Mannheim, University of Heidelberg, Th.-Kutzer-Ufer 1-3, Mannheim 68137, Germany

**Keywords:** Soft tissue sarcoma, O6-methylguanine–DNA methyltransferase, Promoter methylation, Temozolomide, Epigenetic gene silencing, Radiation therapy

## Abstract

**Background:**

Gene silencing of O6-methylguanine–DNA methyltransferase (MGMT) by promoter methylation improves the outcome of glioblastoma patients after combined therapy of alkylating chemotherapeutic agents and radiation. The purpose of this study was to assess the frequency of MGMT promoter methylation in soft tissue sarcoma to identify patients eligible for alkylating agent chemotherapy such as temozolomide.

**Findings:**

Paraffin tumor blocks of 75 patients with representative STS subtypes were evaluated. The methylation status of the MGMT promoter was assessed by methylation-specific polymerase-chain-reaction analysis (PCR). Furthermore, immunohistochemistry was applied to verify expression of MGMT. MGMT gene silencing was assumed if MGMT promoter methylation was present and the fraction of tumor cells expressing MGMT was 20% or less. Methylation specific PCR detected methylated MGMT promoter in 10/75 cases. Immunohistochemical staining of nuclear MGMT was negative in 15/75 cases. 6/75 tumor samples showed MGMT promoter methylation and negative immunohistochemical nuclear staining of MGMT. In none of the tested STS subtypes we found a fraction of tumors with MGMT silencing exceeding 22%.

**Conclusion:**

MGMT gene silencing is a rare event in soft tissue sarcoma and cannot be recommended as a selection criterion for the therapy of STS patients with alkylating agents such as temozolomide.

## Findings

### Hypothesis

Soft tissue sarcoma (STS) form a heterogeneous group of malignant tumors arising in the mesenchymal tissues. A considerable fraction of patients presents with locally advanced disease and requires multimodal therapy including chemotherapy or chemoradiation [1]. Doxorubicin and ifosfamid are generally recommended for first line systemic treatment in unresectable and/or metastatic STS. A variety of other chemotherapeutic substances have been tested in phase II trials but were never compared to standard treatment in phase III trials although some of them may be regarded as active drugs in STS [2]. Temozolomide is an oral prodrug of 3-methyl-(triazen-1yl)imidazole-4-carboximide, the active metabolite of the alkylating agent DTIC [3]. In combination with radiation therapy, temozolomide has substantially improved overall survival in glioblastoma patients and has altered the standard of care in glioblastoma treatment [4]. The activity of temozolomide in glioblastoma patients relies on gene silencing of the MGMT (O6-methylguanine–DNA methyltransferase) DNA repair gene, though [5]. In STS, temozolomide the activity of temozolomide seems to be schedule dependent [6,7]. The combination of temozolomide with irradiation which would be suitable for neoadjuvant treatment of locally advanced STS seems to be well tolerable and reveals promising response rates [8]. Further phase II and/or III trials are necessary to evaluate the efficacy of temozolomide combined with irradiation. The design of these trials must take into account MGMT gene silencing for its impact on the activity of temozolomide. The purpose of the present study was to determine the frequency of MGMT gene silencing in soft tissue sarcoma in order to identify patients and/or histological subtypes that may be suitable for treatment with alkylating agents and irradiation in the setting of a phase II trial.

## Methods

Paraffin blocks of tumor biopsie or resections of 75 patients with representative histological sarcoma subtypes were obtained from the local tumor tissue bank (Table 1). These tumor blocks were analysed by methylation specific polymerase chain reaction (PCR) and immunohistochemistry to detect MGMT gene silencing. Ethical approval was obtained from the Medical Ethics Committee II, University of Heidelberg (Reference number 2006-154N-MA). Written informed consent was obtained from the patient for publication of this report.

**Table 1:** Patient and Tumor Characteristics

**N**	**75**
**Sex**	
Female	33
Male	43
**Age** (Median/range, years)	54 (18–83)
**Size**	
>10cm	43
<10cm)	32
**Grade**	
1	13
2	24
3	38
**Histological subtype**	
Liposarcoma	18
MFH/NOS	15
MPNST	10
Leiomyosarcoma	14
Myxofibrosarcoma	11
Synovial sarcoma	7
**Site**	
Lower limb	21
Upper limb	12
Retroperitoneum	21
Trunk	12
other	9

For methylation specific PCR, genomic DNA was isolated from paraffin sections of the selected tissue blocks and bisulfite converted with a commercial kit (EpiTect Plus FFPE Bisulfite Kit; Qiagen; Hilden, Germany) according to the manufacturer’s instructions. The bisulfite treated DNAs were amplified by a two-step PCR approach to improve the sensitivity of detecting methylated alleles [9]. For the first round of amplification the primer sequences were 5’-GGATATGTTGGGATAGTT-3’ (MGMT-F) and 5’-CCAAAAACCCCAAACCC-3’ (MGMT-R). PCR conditions included an annealing temperature of 52°C and 40 cycles of amplification. For nested PCRs with specific primer sets for un-methylated and methylated samples 1μl of the first round amplification product was used at an annealing temperature of 60° with 35 amplification cycles. Primer sequences of MGMT for the unmethylated reaction were 5’-TTT GTG TTT TGA TGT TTG TAG GTT TTT GT-3’ (MGMT-UUP) and 5’-AAC TCC ACA CTC TTC CAA AAA CAA AAC A-3’ (MGMT-ULP). The sequences of MGMT for the methylated reaction were 5’-TTT CGA CGT TCG TAG GTT TTC GC-3’ (MGMT-MUP) and 5’-GCA CTC TTC CGA AAA CGA AAC G-3’ (MGMT-MLP). PCR products were separated and visualized on a QIAxcel electrophoresis system (Qiagen, Hilden, Germany). As a control we selected several cases of glioblastoma with MGMT promoter methylation and negative control samples without DNA and included both for each set of PCR.

Immunohistochemical analysis of formalin-fixed, paraffin-embedded sections of the selected tumor samples was performed according to standard protocols. The primary antibody used was MGMT Monoclonal Antibody MT 23.2 (Pierce Antibodies, Il USA) after heat antigen retrieval in pH 9 buffer (Dako, Glostrup, Denmark). Only nuclear staining was considered positive. MGMT expression was considered lost when there were 20% or less stained tumor cell nuclei.

MGMT gene silencing was assumed if methylation specific PCR detected MGMT promoter methylation AND immunohistochemistry demonstrated MGMT expression in less or equal than 20% of tumor cells. Results of these analyses are presented descriptively. Median and range are given if appropriate.

## Results

**Figure 1:**
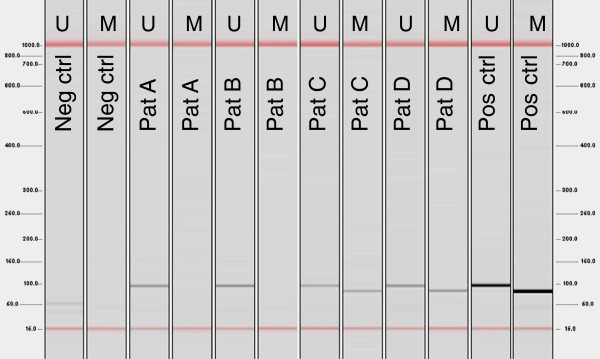
**Example of methylation specific PCR for unmethylated and methylated MGMT Promoter.** U: unmethylated, M. methylated.

Methylation specific PCR detected unmethylated MGMT promoter in 65/75 cases. In 10/75 tumor samples we found MGMT promoter methylation. Yet, in each of these cases also unmethylated MGMT promoter was detected. An example of the methylation specific PCR is given in Figure 1.

**Figure 2:**
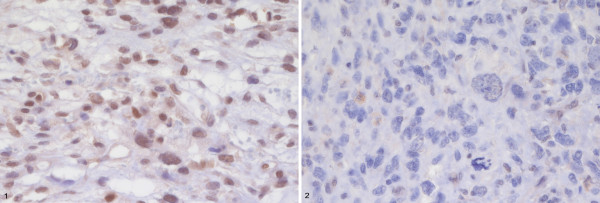
**Example of immunohistochemistry for MGMT in two cases of STS Non Otherwise Specified (NOS).** 1: positive staining, 2: negative staining.

Immunohistochemical staining showed inhomogeneous expression of MGMT in most patients. Immunohistochemistry was regarded negative in 15/75 cases. An example of negative and positive staining for MGMT is depicted in Figure 2.

In 6/75 tumor samples we detected both MGMT promoter methylation and negative staining of MGMT. In none of the tested STS subtypes we found a fraction of tumors with MGMT silencing (MGMT promoter methylation and negative immunohistochemistry for MGMT) that exceeded 22% (Table 2). Of note, MGMT promoter methylation occurred across all sarcoma subtypes, without preference for a specific entity.

**Table 2:** Fraction of tumors with MGMT gene silencing according to tumor subtype

**Histological subtype**	**MGMT promoter methylation**	**Negative nuclear staining of MGMT**	**MGMT gene silencing**
**Liposarcoma**	2/18	3/18	2/18
**MFH/NOS**	1/15	3/15	1/15
**MPNST**	3/10	3/10	1/10
**Leiomyosarcoma**	1/14	2/14	1/14
**Synovial Sarcoma**	1/7	0/7	0/7
**Myxofibrosarcoma**	2/11	4/11	1/11

## Conclusions

The purpose of the present study was to determine the frequency of MGMT gene silencing in soft tissue sarcoma in order to identify STS patients and/or histological subtypes that may be suitable for treatment with alkylating agents and irradiation in a phase II clinical trial with MGMT promoter methylation being a criterion for selection or stratification. The rationale behind the study was that glioblastoma patients with MGMT gene silencing benefit from chemotherapy with the alkylating agent temozolomide in combination with radiation therapy [4].

We selected a set of paraffin blocks of representative STS subtypes and performed MGMT promoter methylation analysis and immunohistochemistry which demonstrated MGMT gene silencing in only 6/75 of the evaluated tumors. Even in those tumors where MGMT promoter methylation was present, we were able to detect tumor cells with non-methylized MGMT promoter. This phenomenon may be explained by intratumoral heterogeneity or by the detection of non-methylized MGMT promoter in tumor infiltrating lymphocytes, blood vessels or normal tissue as described before [5,10]. Furthermore the various qualitative and quantitative methods for the analysis of MGMT promoter methylation may reveal divergent results even if the sequences tested are overlapping or identical [11,12]. That was the reason to perform additional immunohistochemistry of MGMT expression in all cases of this cohort that confirmed expression of MGMT in most tumors.

Two other groups published data on MGMT gene silencing of STS in Asian populations. Kim et al. examined 62 cases of liposarcoma, leiomyosarcoma, malignant peripheral nerve sheath tumor (MPNST), malignant fibrous histiocytoma (MFH) and synovial sarcoma and reported MGMT promoter methylation in 21/62 cases [13]. Kawaguchi et al. studied the methylation of the MGMT promoter in MFH, MPNST and leiomyosarcoma and found MGMT promoter methylation in 10/65 tumor samples [14]. The rate of tumor samples examined after pretreatment was not reported. Despite differences in methods, patient populations and tumor subtypes in the present and the previously published studies, it is obvious that MGMT gene silencing is a rare event in soft tissue sarcoma. Furthermore no histological subtype can be identified that would exhibit MGMT promoter methylation in a relevant fraction of tumors in more than one of the studies.

A selection criterion for a personalized therapy in soft tissue sarcoma should be easy and reliable to assess, discriminate between tumors that are targeted well and positive in a relevant number of patients [15]. Although MGMT gene silencing may increase the effect of alkylating agents given in combination with irradiation, we believe that the frequency of MGMT gene silencing is too low to use it as a selection or stratification criterion in a phase II trial of concomitant irradiation and alkylating agent therapy.

## Competing interests

All authors declared that they have no competing interest.

## Authors' contributions

JJ, FW, PS and PH participated in the design of the study. PH took responsibility for the funding. MH, CS and PS carried out the methylation specific PCR and immunohistochemistry. JJ and MH performed the statistical analysis. JJ drafted the manuscript. All authors read and approved the final manuscript.
